# Antifreeze Proteins: A Tale of Evolution From Origin to Energy Applications

**DOI:** 10.3389/fbioe.2021.770588

**Published:** 2022-02-03

**Authors:** Ghazaleh Gharib, Shaghayegh Saeidiharzand, Abdolali K. Sadaghiani, Ali Koşar

**Affiliations:** ^1^ Faculty of Engineering and Natural Sciences (FENS), Sabanci University, Istanbul, Turkey; ^2^ Sabanci University Nanotechnology and Application Center (SUNUM), Sabanci University, Istanbul, Turkey; ^3^ Center of Excellence for Functional Surfaces and Interfaces for Nano-Diagnostics (EFSUN), Sabanci University, Istanbul, Turkey

**Keywords:** anti-freeze, coating, genetic engineering, energy system, ice binding protein, industrial application

## Abstract

Icing and formation of ice crystals is a major obstacle against applications ranging from energy systems to transportation and aviation. Icing not only introduces excess thermal resistance, but it also reduces the safety in operating systems. Many organisms living under harsh climate and subzero temperature conditions have developed extraordinary survival strategies to avoid or delay ice crystal formation. There are several types of antifreeze glycoproteins with ice-binding ability to hamper ice growth, ice nucleation, and recrystallization. Scientists adopted similar approaches to utilize a new generation of engineered antifreeze and ice-binding proteins as bio cryoprotective agents for preservation and industrial applications. There are numerous types of antifreeze proteins (AFPs) categorized according to their structures and functions. The main challenge in employing such biomolecules on industrial surfaces is the stabilization/coating with high efficiency. In this review, we discuss various classes of antifreeze proteins. Our particular focus is on the elaboration of potential industrial applications of anti-freeze polypeptides.

## Introduction

**FIGURE 1:**
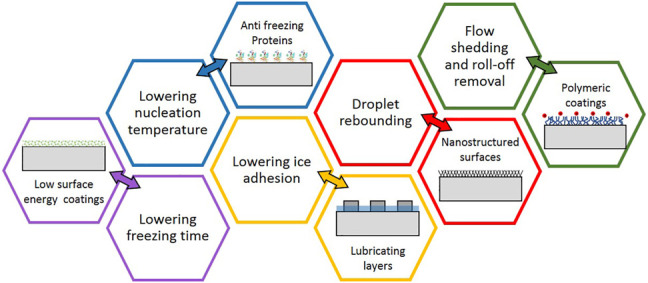
Major passive anti-icing mechanisms with examples.

Ice crystallization and ice formation are major drawbacks that considerably hinder the productivity of industrial machinery and equipment with a cooling system. The ice nucleation activity and freezing particles significantly reduce the system efficiency in a wide range of industrial applications including refrigerators, heat exchangers, wind turbines, helicopters, and aircraft [1]. It is therefore essential to integrate ice preventing mechanisms with the less energy requirement and more efficiency to prevent ice formation at interfaces or elsewhere. The formation of ice introduces excessive thermal resistance as a result of the outer ice layer resulting in deterioration in machine functioning (Moser et al., 2009). On the other hand, accumulated ice layers reduce safety in engineering applications. Slippery pavements, changing the hydrodynamics of the surface of aircraft including wings, airfoils, and rotors, and separating ice layers from wind turbines are examples for human safety issues and catastrophic events. Therefore, rigorous efforts have been made to understand icing and de-icing mechanisms (Lv et al., 2014; Heydarian et al., 2021; Li et al., 2021). Although several anti-icing and de-icing methods and materials have been developed, conventional approaches are often costly, ineffective, and most importantly, are not environmentally friendly (Muthumani et al., 2014). Figure 1 summarizes major anti-icing strategies. In this review, besides elucidating icing and de-icing mechanisms and available de-icing methods, we present a comprehensive discussion on antifreeze proteins and their structural integrity. The possible applications of AFPs in industrial applications are presented at the end of this review.

Anti-icing and de-icing processes can be categorized into passive and active modes. The passive method takes advantage of physical properties of the surface, while the active method relies on external systems, which require an energy supply. Since AFPs are passive agents, this review focuses on passive methods and solutions. In principle, because of their anti-adherent property, ice-phobic coatings prohibit ice from adhering to the surface, whereas super-hydrophobic coatings do not allow water to stay on the surface due to repulsive features (Hao et al., 2014). To minimize the icing problem, many engineered surfaces with topological heterogeneity have been developed and tested (Tarquini et al., 2014). Superhydrophobic surfaces (Kreder et al., 2016a; Stamatopoulos et al., 2017), for example, profoundly reduce the liquid/solid contact area, thereby delaying the onset of heterogeneous ice nucleation. By forming voids between condensate droplets, some of these surfaces result in random droplets bouncing or spatially controlling condensed droplets and hindering the spreading of ice. Another example is lubricant-impregnated surfaces, which can effectively decrease ice adhesion due to their lower elastic modulus and closely packed microscopic crack initiators (Seifert, 2003; Zhao et al., 2021). Biphilic surfaces with high contrast wettability trends also exhibited several benefits, in both slowing the initiation of ice nucleation and promoting ice/frost layer removal (Murphy et al., 2017).

Epoxy or polyester matrix composites reinforced with glass and/or carbon fibers are currently being used by many producers. However, inexpensive polyester and glass fibers are still the material of choice for manufacturers. Current research in chemical anti-icing research is focused on nanocomposite coatings, reinforced particles, and nanometer-scale polymers, which provide high water contact angles (Zhao et al., 2017). Prevention of ice formation by plain coating on operating surfaces is not yet realistic. Several possible materials have been investigated in many laboratories and field experiments in this regard, but no effective and promising solution has been identified (Kreder et al., 2016b). Even on coated surfaces, regardless of the temperature, ice formation could still be observed, and none of the available coatings is practically ice-phobic (Zhuo et al., 2018; Wang et al., 2019). The ice throw, large accretion during extreme icing, and unsymmetric accretion, which contributes to the instability of the blade, are additional disadvantages (Gurumukhi et al., 2020). The coating becomes porous after a short operating time and loses its capacity to prevent icing (Dalili et al., 2009). For many of these coatings, the specific details remain confidential. As a consequence, most datasheets and chemical compositions are categorized as unavailable (Kimura et al., 2003). Most importantly, chemical wastes are contaminants, and special treatment and subsequent maintenance are required. Hence, they cannot be kept on the surface for a long time because of safety concerns (Anderson and Reich, 1997).

## The Origin of Antifreeze Proteins and Their Diversity

A notable diversity within various species of AFP families and their arbitrary placement in the respective phylogeny revealed their evolutionary history back to almost 10–30 million years ago when the sea level began to freeze in Antarctica; however; the degree of expression of ice-binding proteins differs in various species (Seifert, 2003). The investigation based on the origin of the gene families coding various types of AFPs led the scientists to the intriguing fact of their origin from the non-coding fragments of DNA.

Chen and his colleagues concluded that antifreeze linked glycoproteins in antarctic fish are homologous to those in arctic fish, and they were results of evolution through a series of independent molecular pathways from non-coding DNA regions (Tammelin, 2000). According to his theory, the function of the primary version of the AFGP family in notothenioid was limited to antifreeze activity in intestinal fluid; however, later the protein expression was extended to hepatocytes and subsequently released into the circulatory system. There are several speculations regarding their evolution mechanisms, such as exon shuffling, divergence, and duplications of nonfunctional segments of DNA (Dalili et al., 2009).

There are more than 60 identified heterogeneous plant species with AFP activity, where eleven of them have been isolated and characterized by high ice recrystallization inhibition activity (IRI) and low thermal hysteresis. According to these studies, all of the plant-based AFPs evolved from the OsLRR-PSR peptide sequence descendent from almost 36 million years ago (Parent and Ilinca, 2011). The discovery of antifreeze proteins began in the early 70s when Arthur DeVries reported a distinct glycoprotein in Antarctic fish’s blood serum, which helped them to survive at −0.7°C (Chen et al., 1997; Fletcher et al., 2001). Since then, many investigations based on identifications of various AFPs in numerous species conducted by DeVries and his team resulted in interesting findings. The occurrence of the AFP among all categories of teleost species occurred in the Cenozoic era or Antarctic glaciation age, which began 25 million years ago in the Southern Hemisphere prior to Northern Hemisphere. Moreover, a study on structural diversities among various AFPs suggested that the evolution of AFPs from fish species occurred independently in broad-spectrum during the recent Cenozoic glaciation period. Nevertheless, there are numerous types of AFP from different sources, which have the same function in the growth of ice crystals regardless of the difference in polypeptide compositions or the structures (Logsdon and Doolittle, 1997; Gupta and Deswal, 2014).

## Antifreeze Proteins

### Classifications, Types, and Structures

**FIGURE 2:**
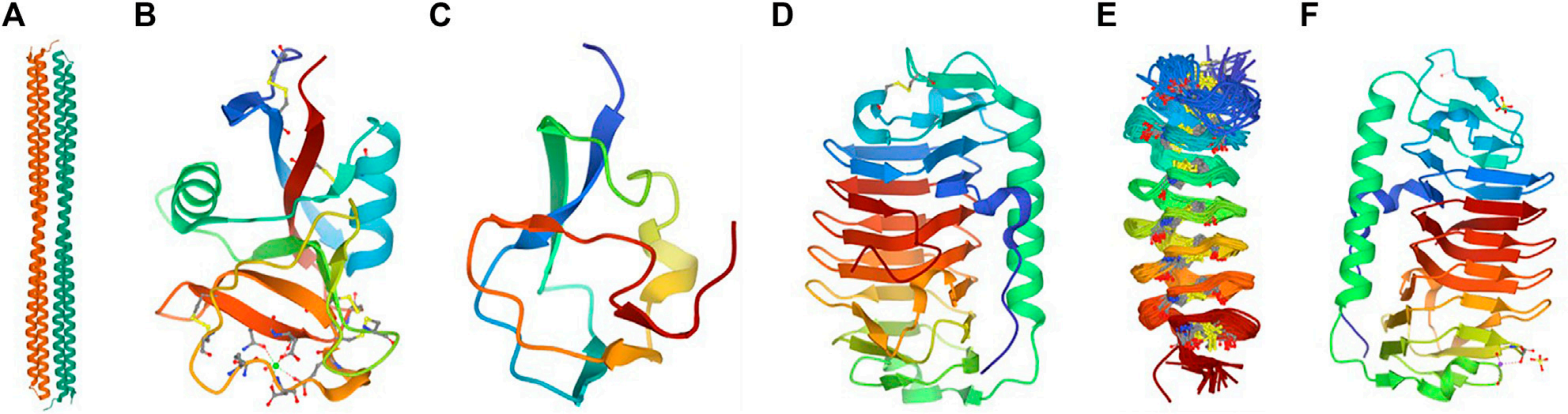
Evolutionary structural formation of antifreezing proteins in various species **(A)** Fish Type I antifreeze hyperactive winter flounder PDB: 4KE2 (Radermacher and Kim, 1996); **(B)** Fish, AFP Type II, *Clupea harengus* PDB: 2PY2 (Moser et al., 2009); **(C)** Fish AFP TYPE III, *Zoarces americanus* PDB:4UR4 (Lv et al., 2014); **(D)** Antarctic sea ice bacterium *Colwellia* sp. PDB:3WP9 (Heydarian et al., 2021); **(E)** Insect cysteine-rich antifreeze protein *Tenebrio molitor* PDB:1L1I (Li et al., 2021); **(F)** Antifreeze protein from a snow mold fungus PDB: 5B5H (Muthumani et al., 2014).

There are three categories of Ice-binding proteins (IBPs). The formation of the ice crystals is controlled by ice-nucleating proteins (INPs) at high subzero temperatures, while the antifreeze proteins (AFPs) decrease the freezing point of body fluids in organisms to avoid freezing. The third category is Recrystallization Inhibition Proteins (RIPs) with insignificant TH activity. While INPs contribute to the generation of ice crystals at the freezing temperature, AFPs effectively mitigate freezing around the organism’s body fluids, and RIPs inhibit the recrystallization of ice in freeze-tolerant organisms that could freeze and yet survive the freezing temperature (Ramløv and Friis, 2020a). According to their primary and tertiary structures, AFPs of antarctic and arctic inhabitants are classified into four groups as I, II, III, and IV (Figure 2). These polypeptides exhibited different structures with essentially the same function, which suppressed the freezing point onset binding with an ice crystal; however, such diverse structures have not descended genetically from the common ancestors (DeLuca et al., 1998).

In a series of structural studies on *Leucosporidium* sp., an arctic yeast, Lee and his team demonstrated that various classes of AFPs from fish (I-IV), plants, and insects shared similar ice-binding domains, which were adequately consistent and hydrophobic; however, the distinct folds in each one were significant (Hanada et al., 2014). These studies also provided sufficient evidence in showing different family species possessing ice-binding properties, which shared the essential common domain of right-handed beta helical structure for ice-binding activity. The structural studies on several AFPs revealed the essential amino acids, which have a fundamental role in the ice-binding process. For instance, threonine and alanine are two primary residues in AFP ice affinity, located on the hydrophobic and likely flat region of the a-helix (Daley et al., 2002). The mutational analysis of threonine residue within the ice-binding site surprisingly demonstrated that the hydroxyl group of these residues has no contribution to facilitate the ice-binding formation, nevertheless the methyl group provides the major support (Cheng et al., 2016) (Table 1). Some properties such as thermal hysteresis activity in species like *Fragilariopsis cylindru*s, a polar diatom’s AFP, are improved in saline environments. As the expression of this multigene family protein increases under cold and salt stress conditions, the microstructural binding of its various isoforms promotes the reduction in the ice freezing point on various ice planar surfaces and consequently protects the diatoms by providing the ambient fluid (Kim et al., 2017a).

**TABLE 1:** Some examples of antifreeze proteins with the known structures. Adapted (Lee et al., 2012).

Organism		Type	Structure / Polypeptide feature
Animals	Fish	I	α helix / Alanine-rich
		II	α, *ß* and loop structure / C-type lectin
		III	Globular cluster / short *ß* strands
		IV	Helix-bundle
		AFGP	Antifreeze glycoprotein
	Insect	*Tenebrio molitor*	β-helix/Right-handed
		*Dendroides canadensis*	
		*Choristoneura fumiferana*	β helix/Left-handed
Microbes	Bacteria	*Marinomona sprimoryensis*	Globulin-like *ß*- twist/Ca^2+^ binding domain (Baardsnes et al., 1999)
Fungi	Yeast	*Leucosporidium* sp.	Dimeric *ß*-helix/right-handed (Bayer-Giraldi et al., 2011)
Plants	Angiosperms	*Perennial ryegrass*	β-roll domain with eight loops of 14–15 amino acids (theoretical) (Kuiper et al., 2001)
	Diatom(Algae)	*Chaetoceros neogracile*	One *a* helix and 7 *ß* loops (theoretical) (Kim et al., 2017c)

**FIGURE 3:**
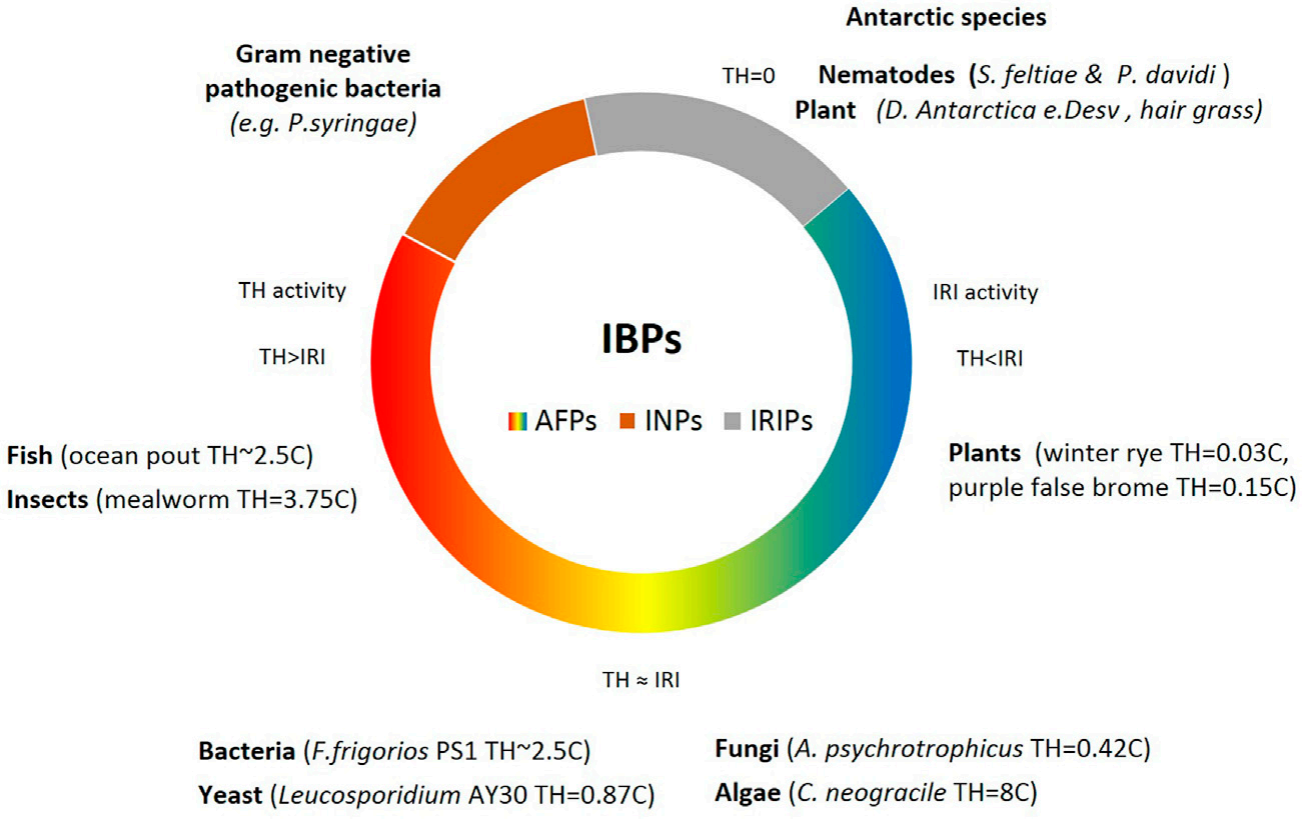
The classification of the IBPs. Antifreezing proteins, AFPs (gradient color), and ice nucleating proteins, INPs, brown color are two categories of Ice binding proteins (IBPs). With the exception of a group of pathogenic bacteria that demonstrate ice nucleating activities, other organisms exhibit antifreezing activities with different degrees of thermal hysteresis and ice crystallization inhibition activities (Hao et al., 2014).

The activity of AFPs in various life forms is determined by two main elements: Thermal Hysteresis (TH), and Ice Recrystallization Inhibition (IRI) phenomenon. Insects and fish exhibited high TH with no or mild IRI activity. Bacterial and similar microorganisms demonstrated adequate amounts of both IRI and TH; however, in contrast, plants demonstrated low TH and high levels of IRI. The excretion of AFPs in a substantial amount helps various microbes to endure the sub-zero condition, and the AFP secretion assists them to gather sufficient nutrients and oxygen (Jia and Davies, 2002). Since AFPs are subcategories of IBPs, we could conclude that all AFPs have IBPs functions, but not all IBPs are antifreeze proteins. Nonetheless, many scientists could use them conversely (Guo et al., 2017) (Figure 3). Interestingly, the intracellular ice recrystallization inhibition activity was observed in various cryophilic species of nematodes including *S. feltiae* and antarctic *P. davidi* with no thermal hysteresis activity (Wharton et al., 2005). The intracellular IRI proteins in these species control the formation of ice crystals in the cells equipped with the specialized cell membrane by maintaining small ice crystals. Recrystallization inhibition (RI) activity prevents small ice crystals from forming larger ones that could be lethal at −3°C (Ali and Wharton, 2014). The IRIPs were extensively characterized from versatile sources of plants or animals living under extremely harsh conditions such as freeze tolerance Antarctic hair grass, *Deschampsia E. Desv* (John et al., 2009), and Wood frog *Rana sylvatica* (Le Tri et al., 2019), which survive at −30 and −16°C, respectively.

### Mechanism of Amino Acid Interaction in Antifreeze Proteins With ice Lattice

The initial hypothesis was based on the computational analysis proposed by Chou in 1992. This mechanism suggested that upon the protein interaction with the ice lattice, the complex structure reaches the lowest optimum energy level, which is the most favorable position for the polypeptide chain. In this simulation, the helical structure of the winter flounder antifreeze protein consisted of multiple threonine residues (located in positions 2, 13, 24, and 35) such that their hydroxyl groups were aligned in parallel and proximate distances with an axis in the direction of the ice. According to this hypothesis, such an arrangement allows the polypeptide chain to bind in a zipper-like configuration with ice lattice *via* the hydrogen atoms present in threonine side chains with oxygen atoms towards the ice lattice; thereby suppressing the freezing point and eventually preventing further enlargement of planes in the pyramidal of the crystal ice (Lee et al., 2012). However, the recent analysis of molecular dynamics (MD) simulation and the crystal structure of the same protein, winter flounder fish AFP, suggested a new theory.

Cheng and Merz proposed a mechanism, which explained the clathrate hydrate generation on the protein surface resulting in hydrogen bonding and hydrophobic effects, which subsequently reduced the dissolved solution’s free energy (Białkowska et al., 2020). Hence, among the two bonds involved in the clathrate interface, AFPI interactions were more stable due to thermodynamically favorable outcomes; therefore, it was more likely to suggest that the initial protein bonded to the encountering pre-arranged ice surface (Kim et al., 2017a). There are three possible ice-binding theories: 1) A hydrophobic flat region of the protein fits within the ice by directing its hydrophobic side chain entangling inside the ice lattice; 2) The ice lattice oxygen atoms engage with the hydrogen bonding residues on the exterior zone of the ice-binding proteins, and 3) Formation of the side binding network on the surface water supported by the protein residues (Białkowska et al., 2020).

### Natural Sources and Genetically Engineered AFPs

Several transgenic plants such as *Nicotiana tabacum* and *Arabidopsis thaliana* are designed to produce AFPs from cold-loving species of Winter flounder (Chou, 1992) and Fire-colored beetle (Cheng and Merz, 1997), respectively. These functionally active proteins expressed with signal transport sequence of hydrophilic multi-domains with an ice-binding activity and then stored in the apoplast act remarkably in inhibiting both ice nucleation and ice crystallization in plants (Oude Vrielink et al., 2016).

The needle-like ice crystals are often generated from highly concentrated AFP (1 mg/ml) and perform remarkably to promote the process of cell ablation in cryotherapy (Madura et al., 2000). Moreover, the production of various types of genetically engineered AFPs from cold living fish is notable in the cryo-industry, and they are mainly used for food preservation in the freezing process (Kenward et al., 1993). Expressed AFPs from the yeast species of *Leucosporidium* sp. are employed for red blood cell cryopreservation (Huang et al., 2002).

## Anti Icing Mechanism of AFPs

**FIGURE 4:**
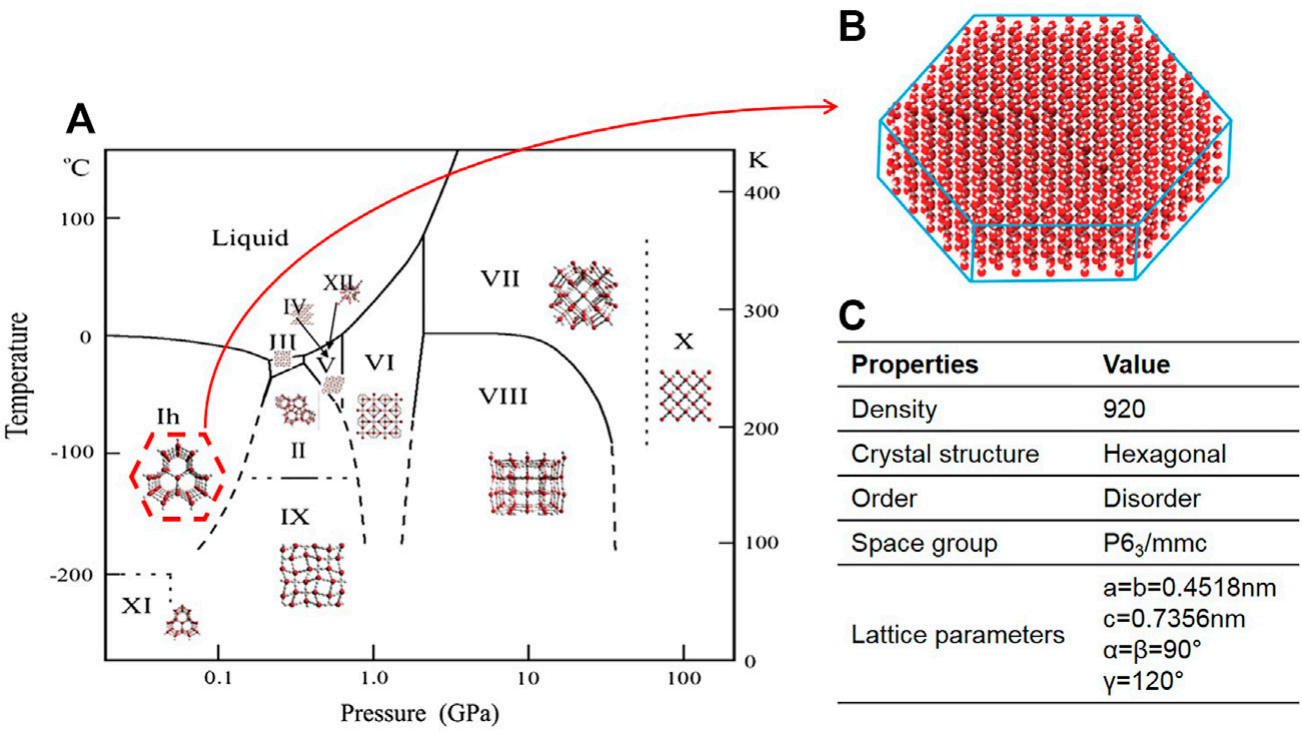
**(A)** phase diagram of ice/water; **(B)** Atomic structure of Hexagonal ice; **(C)** Properties of hexagonal ice (Tarquini et al., 2014).

**FIGURE 5:**
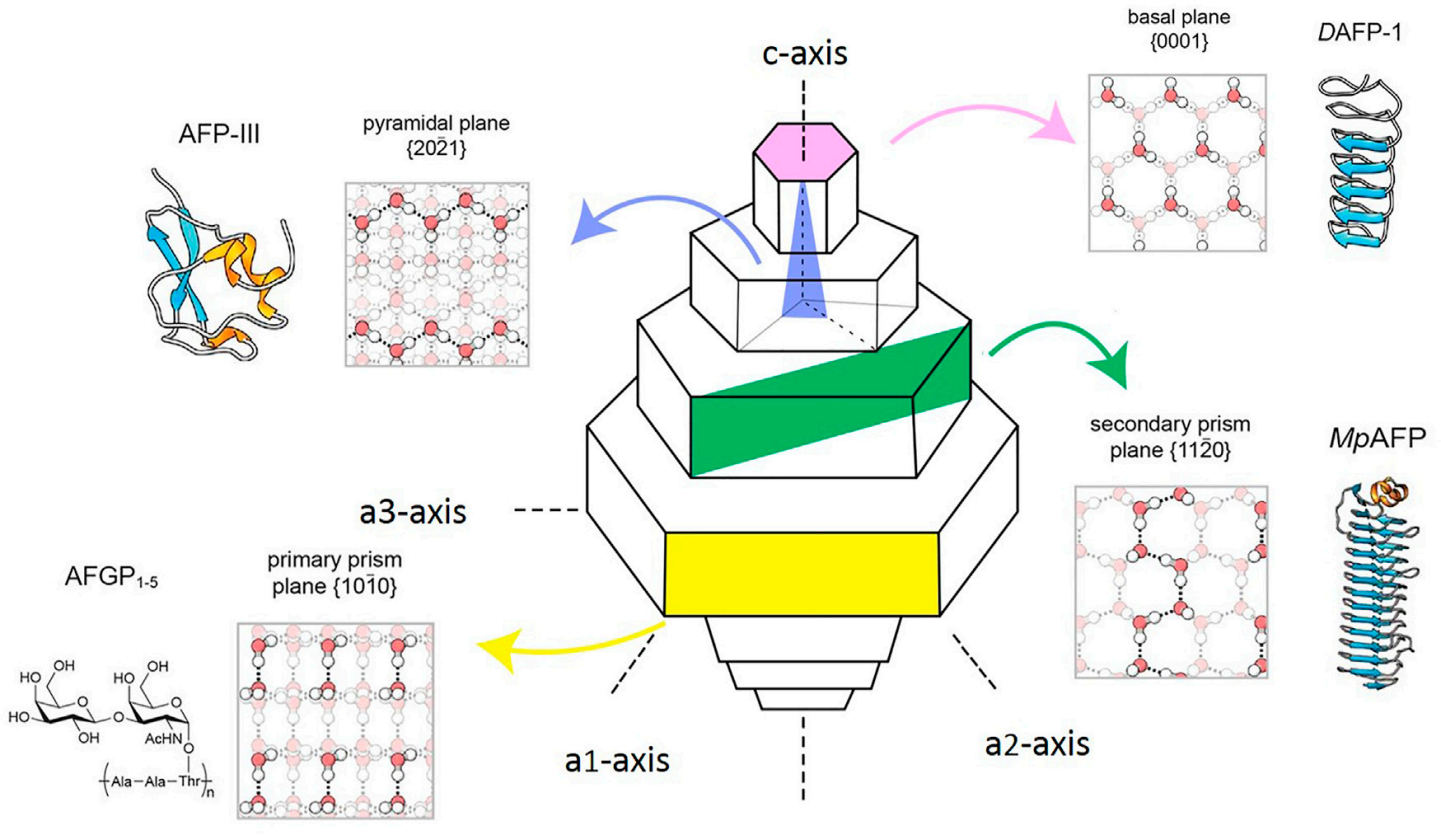
Types of AFP binding on ice planes and water network formation in various species. Different colors represtent the type of AFP adsorption on ice planer.
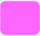
Basal plane e.g. DAFP-1, beetle *Dendroides canadensis*;
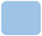
Pyramidal plane e.g., ocean pout, *Zoarces americanus* AFP-III;
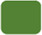
Secondary prism e.g., MpAFP (M. primoryensis);
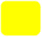
Primary prism plane e.g., AFGP various species of Antarctic Notothenioid fishes; Hydrogen bonding shown in dash lines, white circles; Red circles represent oxygen atoms, white circles represent hydrogen atoms (Seifert, 2003; Stamatopoulos et al., 2017).

The pressure and temperature of the icing course significantly affect its crystallization behavior. 15 types of ice polymorph with densities ranging from 920 to 1,290 kg/m^3^ exist and have hexagonal, cubic, rhombohedral, tetragonal, monoclinic, and orthorhombic crystal structures (Figure 4A). Hexagonal ice (Ih) is the most general form of ice available on Earth (Figures 4B,C) (Griffith and Yaish, 2004). Behind the survival secret of many bacterial species during frozen seasons lies the expression of AFPs, which shields them from growing external ice (Westh et al., 1997). Although the structures of AFPs vary in different organisms, they exhibit various ice growth patterns based on the ice plane features. Figure 5 represents four distinct features of the ice absorption pattern, including the fast-growing pattern that contains the primary prism plane in AFGP of polar fishes; the secondary prism pattern in Antarctic bacterium *M. primoryensis* MpAFP, and the pyramidal plane pattern in AFP type III. However, in some species such as beetle *Dendroides Canadensis*, the AFP demonstrated the basal plane pattern with slower ice growing pattern as compared to others. Accordingly, the growth rate of the ice crystals is directly proportional to the growing length of the hydrogen bond of water molecules on the surface of the ice plane (Sander and Tkachenko, 2004). Inhibition of recrystallization is another function of AFPs. The recrystallization makes water molecules travel and build up the largest ice crystals with smaller surface areas with greater stability. Eventually, these enormously formed ice crystals are the cause of major harm to living organisms. AFPs are found in almost all organisms residing at subzero temperatures, where ice recrystallization is life-threatening (Zachariassen et al., 1999).

The organisms could use the ice-binding affinity of AFPs for various purposes. The polar fish uses these proteins to inhibit the internal ice recrystallization and growth of crystals. While the algae uses it as a tool for building the ice in specific orientations, an Antarctic bacterium *Marinomonas primoryensis* exerts IBPs for the means of ice surface adhesion (Xu et al., 1998). The Arctic habitat of the same species possesses multi-domain AFPs to retain the nutrients and oxygen *via* the Ca2+-dependent ice-binding site of the adhesive protein. This structure may delay the freezing point by more than 2°C. Many organisms, including various species of bacteria, algae, fungi, and yeast, endure in a frozen biosphere with the virtue of extracellular ice-binding domains, which help them proliferate within hydrated microchannels (Knight and Duman, 1986).

Antifreeze proteins function distinctively towards ice-forming solutions. The AFPs decrease the solution’s freezing point to a level lower than the ice melting point, which is known as thermal hysteresis. AFP acts as a cushion to equilibrate the freezing temperature with the ice growing point. In the mixture of the aqueous solutions, with gradually decreasing temperatures, water molecules form more ice crystals and therefore elevate the concentration of the surrounding solutes. Once the fluid vapor pressure equilibrates with that of the crystal fractions, the surrounding temperature becomes equal to the melting point of the ice crystals. The presence of antifreeze proteins in the solution hinders ice growing, which eventually causes a temperature drop in the surrounding fluid (Ramløv and Friis, 2020b). When AFP binds to the growing side of the ice crystal, it causes the crystal to stop growing until the fluid temperature reaches the freezing point then it keeps growing further under a defined plane. The ice-binding affinity of AFPs occurs at the level of the microstructure. Such proximity of surface-surface interactions within protein polypeptides core complements the lattice structure of the ice crystal in 3D dimensional spatial form. The resultant optimization derived from AFP bound ice crystal structure affects the entropy in reducing the Gibbs free energy (Raymond and DeVries, 1977).

### Ice Nucleation and Growth

At the ice nucleation stage for undisturbed pure water, the supercooled liquid experiences temperatures lower than equilibrium and forms ice crystals. Here, spherical ice embryo growth in the liquid phase decreases the entropy, which eventually creates a hurdle against further nucleation. As a result of the hurdle of the barrier formed by critical free energy, the embryo radius in the medium becomes inversely proportional to the free energy barrier. The embryo radius of ice in the water medium is approximately 10 nm. The critical Gibbs free energy barrier is given as:

$$\Delta G_{f}^{*}=\frac{16\pi \sigma_{\text{iw}}^{3}}{3\Delta G_{\text{f,v}}^{2}}$$ (1)

According to the classical nucleation theory, entropy minimization is the necessity for the formation of spherical ice embryos in the liquid phase. The required change in Gibbs energy for the formation of ice embryos containing $n_{k}$ water molecules ( $\Delta G_{k}$ ) forms an energy barrier to ice nucleation. This energy barrier consists of bulk and surface terms as follows:

$$\Delta G_{k}=\underset{\text{Bulk}\;\text{term}}{\underset{︸}{n_{k}.\left[\mu_{i}\left(T\right)-\mu_{w}\left(T\right)\right]}}+\underset{\text{Surface}\;\text{term}}{\underset{︸}{4\pi r_{e}^{2}\sigma_{iw}\left(T\right)}}$$ (2)

Here, $n_{k}$ , $\mu_{i}$ , $\mu_{w}$ , $r_{e}$ , and $\sigma_{iw}$ are the number of water molecules in the ice embryo, the chemical potentials of ice and water, the radius of ice embryo, and the surface tension between ice and water, respectively. The bulk term (first term on the RHD of Eq. 2) explains mitigation in chemical potential experienced by the embryo. Similar to other phase change processes, the minimization of free energy is the propulsion for ice formation, where the nucleation of the ice is due to the lower chemical potential of the crystalline plane compared to the ambient (*μ*_i_ < *µ*_w_). The subcooling temperature initiating the process is correlated with the chemical potentials as follows:

$$\Delta \mu =\mu_{w}-\mu_{i}=\frac{\Delta H_{m}}{T_{m}}(T_{m}-T)=-k_{B}T\ln\left(\frac{e_{sw}\left(T\right)}{e_{si}(T)}\right)=-k_{B}T\ln S_{i}$$ (3)

Here, $\Delta H_{m}$ , $T_{m}$ , $k_{B}$ , $e_{sw}$ , $e_{si}$ , and $S_{i}$ are the molecular enthalpy of melting, the melting temperature, the Boltzmann constant, the saturation vapor pressure over water and ice, and the ratio of the saturation vapor pressure over water and ice, respectively. The volume ratios of the embryo to water molecule give the number of water molecules as $n_{k}=\frac{4\pi }{3}\frac{r_{e}^{3}}{\nu_{ice}\left(T\right)}$ . Here, $\nu_{ice}\left(T\right)$ can be calculated from the ice density. Rearranging Eq. 3 leads to the following expression:

$$\Delta \mu =n_{k}\left(\mu_{w}-\mu_{i}\right)=-\frac{4\pi }{3}\frac{r_{e}^{3}}{\nu_{ice}\left(T\right)}k_{B}T\ln S_{i}$$ (4)

The surface term in Eq. 1 ( $4\pi r_{e}^{2}\sigma_{iw}\left(T\right)$ ) defines the mandatory energy for the formation of the embryo interface. As can be seen, a spherical ice embryo with macroscopic interfacial tension is assumed for the analysis. The maximum energy barrier is obtained at the critical ice embryo ( $r^{*}$ ) as follows:

$$r^{*}=\frac{2\nu_{ice}\sigma_{iw}}{k_{B}T\ln\left(\frac{e_{sw}}{e_{si}}\right)}=\frac{2\nu_{ice}\sigma_{iw}}{k_{B}T\ln S_{i}}$$ (5)

The barrier energy can be found by integrating the obtained expressions for bulk and surface terms as follows:

$$\Delta G^{*}=\frac{16\pi }{3}\cdot \frac{\nu_{ice}^{2}\sigma_{iw}^{3}}{{\left(k_{B}T\ln S_{i}\right)}^{2}}$$ (7)

In reality, the presence of external bodies reduces the nucleation barrier by promoting the ice nucleation *via* lowering the interfacial free energy (surface term in Eq. 2). This process is defined as heterogeneous nucleation. The quantitative effect of external bodies on the nucleation barrier can be characterized by a correction factor ( f(m,r') ). The correction factor represents the foreign body induced nucleation barrier reduction, and is defined as (Liu et al., 1997):

$$f\left(m,r'\right)=\Delta G_{Hetero}^{*}/\Delta G_{Homo}^{*}$$ (8)

Here, $m=\left(\gamma_{sf}-\gamma_{sc}\right)/\gamma_{cf}$ is the interfacial free energy difference and r'=r/r∗ is the non-dimensional radius. γ_sf_, γ_sc_, and *γ*_cf_ are the surface-fluid, the surface-crystal, and the crystal-fluid interfacial free energies, respectively. The strength and structure of the interaction between crystal and surface alter the interfacial free energy difference (m). The interface geometry also affects the correction factor ( f(m,r') ). The effect of interface geometry can be examined using the Gibbs-Thomson effect.

**FIGURE 6:**
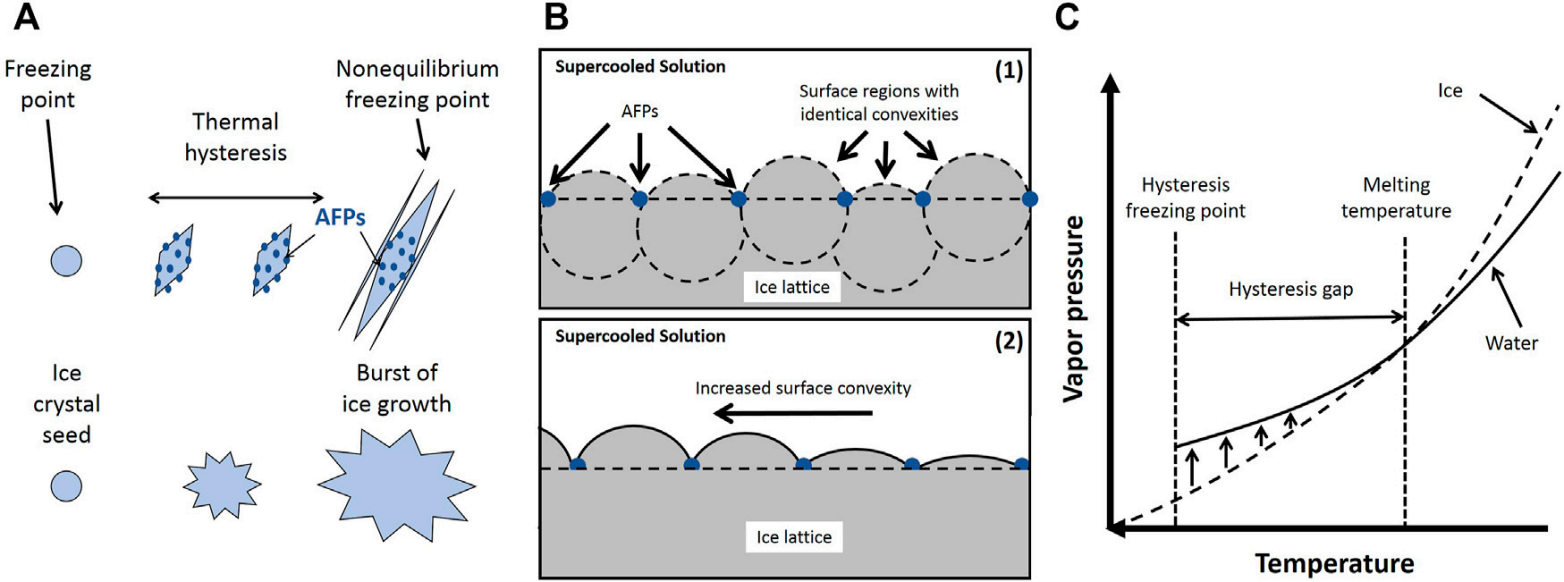
**(A)** Schematic of icing with and without AFPs; **(B)** The convexities’ of the growth zones; **(C)** Equilibration of vapor pressure within a temperature interval near the melting temperature. Adapted from Chen and coworkers (Zhao et al., 2021).

The Gibbs-Thomson effect refers to a thermodynamically unstable state at a curved interface due to an increased vapor pressure or chemical potential. At this state, additional driving forces such as lower supercooling temperature (lower freezing point) are needed to reach the thermodynamic stability at the curved interface. As stated before, AFPs activity is attributed to their binding to the specific surfaces on the ice seed crystals, which causes modification in ice embryo growth morphology. These proteins prevent the growth of the ice crystals into the water by creating a layer around the solid ice. The adsorption-inhibition theory is the most accepted mechanism for the freezing point reduction and ice formation inhibition of AFPs (Kim et al., 2017b). This theory proposes the compact binding of IBPs to the surface of ice crystal molecules which restrict the ice expansion only within the defined borders protein residues (Figure 6A **)**. This confined affix results in a greater surface curvature, as the ice enlarges, subsequently leading to lowering the freezing point caused by the Gibbs-Thomson effect, and ultimately obstructs the ice formation (Figure 6B). When the absorbed AFPs surface curvature radius reaches a critical radius at any melting temperature below the normal, the further formation of curved surface expansion is ceased. This phenomenon results in the formation of spherical growth regions on the ice crystal surface by identical convex shapes, i.e., similar regional vapor pressures. Thus, the entire surface of the ice crystal becomes equal to the vapor pressure of the neighboring supercooled mixture, and later the crystal surface reaches the melting point (Figure 6C).

**FIGURE 7:**
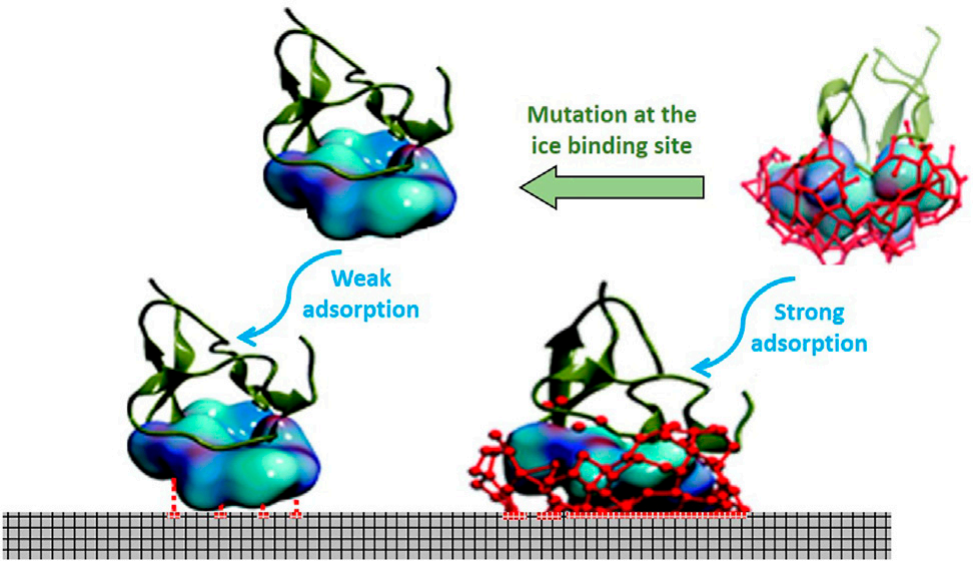
The mechanism action of the wild vs. mutated type III AFP adsoption on ice surface. The IBS of the protein contains hydrophobic residues that drive water molecules (blue) to arrange in the form of hydration complex (red sticks). The similar synergy between water molecules of the formed hydration shell with water molecules on the ice plane (gray squers) causes further adhesion of AFP to the ice surface (Kreder et al., 2016a).

Chakraborty and Jana recently demonstrated the mechanism of AFP adsorption on the ice surface by mutational analysis on type III AFP (Chakraborty and Jana, 2019). Based on this study, the protein consisted of the flat and hydrophobic surface as an ice-binding surface (IBS). Once the temperature dropped, the hydrophobic hydration force of these residues brought water molecules to generate an impact organized structure of the hydration shell on the ice-binding surface. This cage comprised of arranged water molecules, which possessed a similar synergy with the rest of the water molecules of the ice surface and thus made AFP further remain attached to the ice surface. The residual mutation of the protein-binding site distorted the hydration complex, and as a result, the binding affinity of protein towards the ice surface was drastically mitigated. The hydrophobic force of the hydration layer plays a significant role in the absorption that cannot be compromised with the hydrogen bonding within the water molecules. The mechanistic insight of the hydration complex in wild and mutated AFP is represented in Figure 7. A recent study on the structure of an ice-binding protein from a psychrophilic fungus, *Antarctomyces psychrotrophic,* revealed that the protein structure with 6-ladder *ß*-helices was responsible for generating a remarkable water network, which formed a well-tuned binding. This network was supported by a particular side of hydrophobic residues within AFP polypeptide and plans of ice crystal prism (Meister et al., 2019).

In similar studies on AFP type I isolated from winter flounder, the association of AFP with ice does not happen directly but with the conserved hydrophobic aminoacids interface ice-water regions. Following the adsorption, the energy derived from the ice/water interface is drastically reduced, and the protein is attached to the ice side (Voets, 2017). Such a binding affinity forms adequate proximity between the protein helical structure and ice interfacial region, a complex of kinetic pinning, which guarantees the antifreeze activity (Gilbert et al., 2005). The contribution (of both enthalpic and entropic) in the antifreeze mechanism, however, remains with an only trivial amount of excessive free energy to maintain the orientation of the relevant protein with ice/water interface (Yamauchi et al., 2020). Nevertheless, in some cold-tolerant microorganisms such as plant pathogenic bacteria, *Pseudomonas syringae*, the ice nucleation activity is employed as the heat resource. The resulting surface with formed ice crystals from INPs activity acts as a shield against harsh temperatures of −5 to −12°C. In addition, these pathogenic species take advantage of the wound created from ice crystals by absorbing the nutritions from the protective cell layer of Pythium in host vegetables or fruits (de Araujo et al., 2019).

Since water possesses higher vapor pressure than the bulk of ice, the net conversion of the water molecules to form ice occurs at a temperature below the melting point and therefore results in growing ice crystals. However, AFP-ice crystal bounds keep the temperature intermission unchanged which results in vapor pressure equilibration between ice and water in all temperatures that exist in the gap. This is due to the fact that the rate of water molecules adding to the surface of the crystal is equal to those fleeing away, otherwise, the net transport of water particles would be from the solution to ice or vice versa, and it changes the crystal amount. Evidently, the function of AFPs is not based on lowering the water vapor higher than the other effective solutes (Westh et al., 1997). The discrepancy between the water vapor pressure and that of the ice increases when the temperature reaches to a level lower than the equilibrium ice-to-water phase change temperature. Therefore, the ice vapor pressure affected by AFPs is solely reflected by increasing or decreasing the temperature.

Due to the mechanism of thermal hysteresis, ice crystals bearing AFP cannot obtain the equilibration with their environment temperature inside the hysteresis gap. Thus, below the hysteresis temperature points, the AFPs continue interacting with the ice even though the ice fraction severely raises. Moreover, the water molecules in the ice are dynamic, and there is a surface tension gradient induced water molecule flux from small ice embryos toward bigger ones. The flux is conducted through the liquid phase, and at this interface, AFPs can affect systems with essentially frozen solid bases. By covering the variations in surface tension between ice embryos, the molecular flow and equilibration are stopped or significantly reduced.

### Intensity of ice Nucleation

The intensity of ice nucleation can be used to characterize the anti-icing activity of AFPs. The Gibbs free energy can be transferred in the form of nucleation rate, which can be utilized as a criterion to characterize the intensity of ice nucleation. Accordingly, the nucleation rate (J), defined as the number of nucleation per time per volume, can be calculated as:

$$J=A\exp(-\Delta G^{*}/k_{B}T)$$ (9)

**FIGURE 8:**
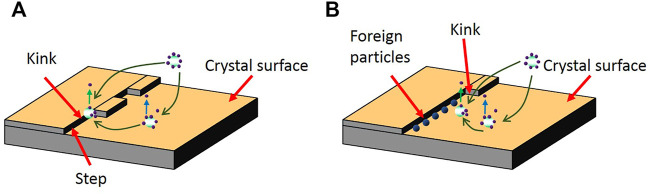
**(A)** Crystal molecules enter kink sites on the crystal surface during the nucleation; **(B)** Suppression of the crystal molecules’ movements towards the crystal surfaces by the absorbed external particles in the kink sites [adapted from (Murphy et al., 2017)].

Here, A and $\Delta G^{*}$ are kinetic pre-factor and Gibbs free energy for ice nucleation, respectively. The ice nuclei can also absorb the foreign particle or additive molecule, which interferences with the conjunction of crystals and the surface of the nuclei kink sites (Figure 8A). The continuation of crystal nucleation requires the removal of foreign bodies from these sites. The β_kink_ parameter can be used to characterize the kinetics of retardation of ice nucleation process as follows (Liu, 2001):

$$\beta_{kink}∼\exp(-\Delta G_{kink}^{\#}/k_{B}T)$$ (10)

$\Delta G_{kink}^{\#}$ is the barrier for the removal of the absorbed external particles from kink sites. AFPs can hinder ice nucleation by occupying the kink sites and increasing the kink barrier (Figure 8B).

Some antifreeze proteins such as RmAFP, an Insect Rhagium mordax, exhibited distinctive behavior of retaining the newly absorbed water molecules descended on the surface of the ice. The direction of the hydrogen bond formation and water molecule differs from the ice structure; therefore, it prevents the addition of further water molecules interfacing the ice surface and protein (Kutschan and Thoms, 2016). Investigations on various structures of antifreeze proteins (AFP) from *pseudomonas* species exhibited high identity amino acid sequences with those of the ice-nucleation proteins (INP) from the same species (Muryoi et al., 2004). The conserved sequence is observed at the location, where the protein binds to the ice in both categories. Nevertheless, the AFP domain displayed higher hydrophobicity than the INP, representing a different mechanism of the protein interaction with ice in two proteins (Sun et al., 1995).

## Applications

### AFPs Coating

The current applications of APFs are majorly focused on agriculture, food, and cryomedicine industries. AFPs are promising supplements for frozen food (Ramløv and Friis, 2020c) including ice cream (Warren et al., 1992; Clarke et al., 2004), meat (Payne et al., 1994; Payne and Young, 1995), frozen dough (Jia et al., 2012; Zhang et al., 2015) and fruit and vegetables (Kong et al., 2016; Kong et al., 2017). There exist several successful applications of AFPs in defrosting food (Ding et al., 2015). In this section, we exploit further possible industrial applications for AFPs. The AFPs can be applied to the surface *via* different coating methods such as electrodeposition, dip coating, or spray coating. As mentioned earlier, AFPs are classified based on Thermal Hysteresis (TH) and Ice Recrystallization Inhibition (IRI) activity. In thermal applications such as refrigeration, even small changes in the inception temperature of the phase change process affect the system efficiency and energy output. Therefore, AFPs with higher thermal hysteresis (TH) are desired. On the other hand, in turbomachinery applications such as air intake or turbine blades, it is important to maintain small ice crystal sizes. Thus, it is recommended to use AFPs with greater Ice Recrystallization Inhibition (IRI) activity.

There are several recent methods recommended for surface coatings of various biomaterials including antifreeze proteins (Table 2). Polyphenols and phenolics, the main compositions of plant tissues having a broad spectrum of chemical and physical properties, are one of the studied compounds. Tannic acid (TA) and its derivatives are proven to be effective for biocoating applications. The complex formation of TA’s galloyl groups and protein’s amino groups resulted in a promising biocompatible adhesive material (Sileika et al., 2013; Jeong et al., 2018). Moreover, the bioconjugation technique *via* biomaterials such as polydopamine (PDA) (3-glycidoxypropyl) methyldimethoxysilane (GOPTS), and biopoly amino acids such as poly l lysine (Niazi et al., 2021) or metal bonding repeating polypeptides (Brown, 1997) are also known to be efficacious methods. There are many investigations on metallic binding poly peptides, which are predominantly used for coating antifreeze proteins. Aluminum binding peptides (Naik et al., 2002) are the most popular ones in this regard.

**TABLE 2:** Summary of the available AFP coating or engineering surfaces.

References	Coating method/surface
Gwak et al. (2015)	Bioconjugation of Aluminum-binding peptide (ABP) with antifreeze proteins from Antarctic marine diatom for metal coating purpose
Liu et al. (2016)	Incorpration of a hyperactive insect AFP from the beetle Microdera punctipennis dzungarica (MpdAFP) into polydopamine (PDA) and (3-glycidoxypropyl)methyldimethoxysilane (GOPTS) to prepare an ice-binding face (IBF) and non-icebinding face (NIBF) for an anti-icing coating on silicon surface
Esser-Kahn et al. (2010)	Conjugated AFP-polymer were immobilized *via* commercially glass slides with coated aldehyde groups
Ejima et al. (2013)	Novel organic polymer of polyphenol Tannic acid meld with Fe^III^ followed by pH change from acid to alkali results in TA–Fe3+ octahedral complex that can adhere to many diverse solid substrates
Jeong et al. (2018)	TA coating on solid substrates and later AFP immobilization on aluminum surface. The Al surface was treated with a mussel-inspired polymer, polydopamine (pDA)
Zuo et al. (2005)	Novel peptides capable of binding to aluminum and mild steel to protect them from deterioration. Among 12 candidate peptides, VPSSGPQDTRTT was chosen as the best peptide showing fourfold intensity comparing to other peptides, in interaction with metal surfaces
Scotter et al. (2006)	Development of recombinant ABP-Cn-AFP_G124Y_ to overcome protein denaturation and reduction in antifreeze activity
	The improvement of protein stability by using convenient sugar-coating steps. A successful immobilization on Aluminum coated surface using ABP-Cn-AFP_G124Y_ fusion proteins
	Stable antifreeze activity up to 12 days at room temperature
Brown, (1997)	Selection of the repeating polypeptides consisting of 14–28 identical residues with metal binding capacity to gold or chromium among five million various polypeptides
Naik et al. (2002)	Biosynthesis of Ag nanoparticles using metal binding peptides which are able to associate to metal cluster using “memory effect” factor
Niazi et al. (2021)	Employing Poly l lysine for microbial biocoating on metal surfaces resulted in an energy conservation for more than 10%

### Wind Turbines

Wind turbines operate at high altitudes and frequently experience icing on their blades. Local climate and height of turbines are important parameters affecting the frequency of ice formation on the leading edge of their rotating blades. Not only safety hazard (ice detachment from the blades) is an important issue but the presence of ice during the operation also negatively affects the blades and results in an increase in power loss (up to 50% annularly) and mechanical failure (due to vibration and load increase). Additionally, measurement sensors and devices including temperature sensors, wind vanes, and anemometers are influenced by ice. For example, the wind speed can be miscalculated with a large error margin of 60% due to this effect (Chakraborty and Jana, 2019).

**FIGURE 9:**
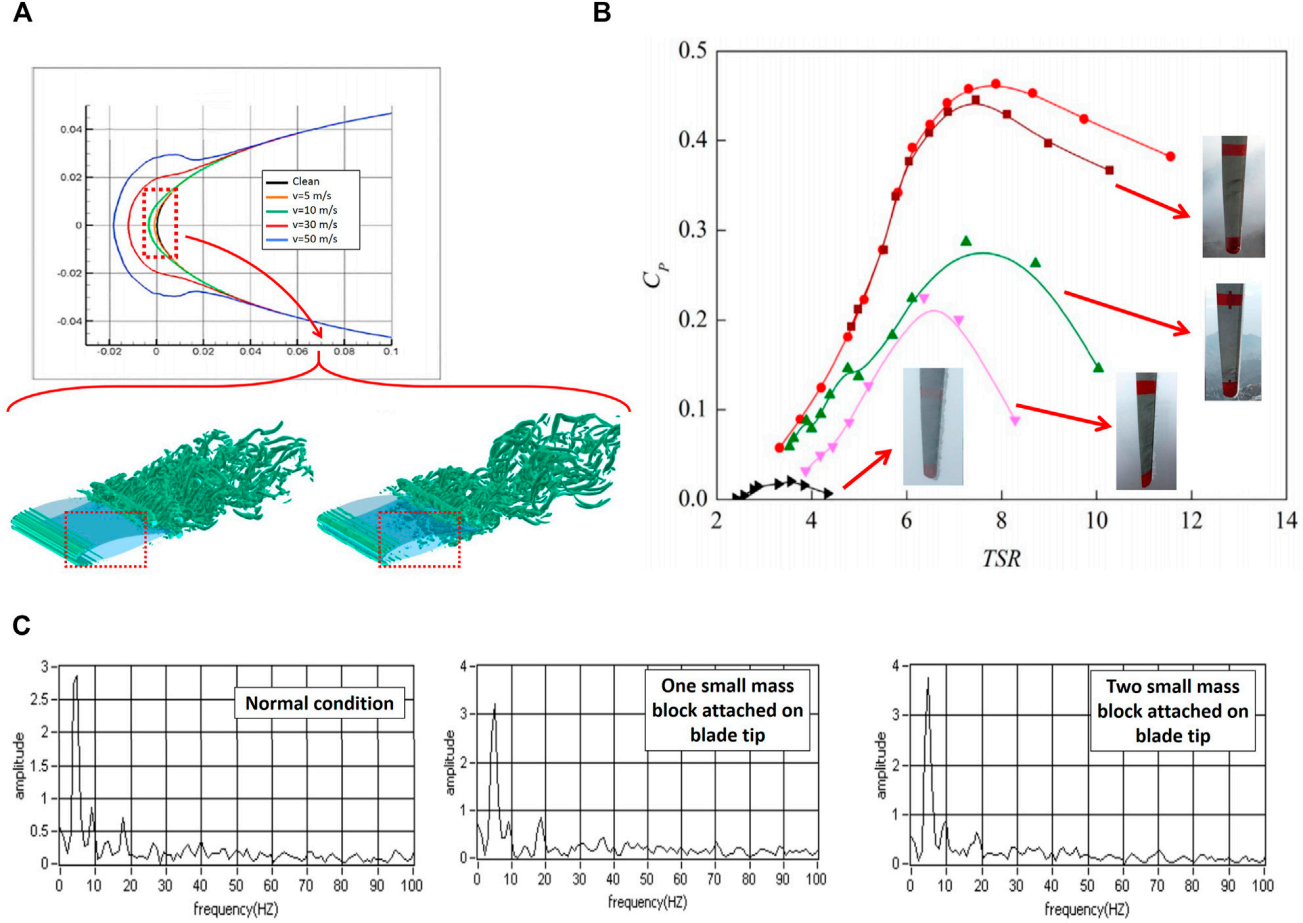
**(A)** Airfoil profile change due to ice accumulation; **(B)** Drag coefficient increase in the wind turbine blades; **(C)** Power amplitude picks for the normal and accumulated blades (Zhao et al., 2017) (Zhao et al., 2017) (Kreder et al., 2016b).

Figure 9A shows the change in the airfoil geometry due to ice accumulation at different wind speeds. As seen, due to a reduction in the ambient temperature, the thickness of formed ice layers increases dramatically (up to 75%) on the airfoil surface. By acting as the surface roughness elements, ice layers change the flow characteristics over the airfoil. In the presence of ice accumulation, the boundary layer thickness increases and grows faster. As a result, the lift to drag coefficient ratio reduces remarkably in icing situations (Figure 9B). Furthermore, the presence of additional ice accumulations on the blade changes the amplitude of the natural frequency of the blade. As shown in Figure 9C, the amplitude of the power frequency vibration increases with the ice layer. The additional mass affects the control mechanism by giving the rotor additional speed when moving from the high point towards the low point (gravity). Furthermore, ice (unbalanced mass) reduces the power output by 1) increasing the inertia moment and causing a delay in the rotor response to the wind speed change, and 2) introducing additional wind power to supply the required kinetic energy.

### Heating, Ventilation, and Air Conditioning

**FIGURE 10:**
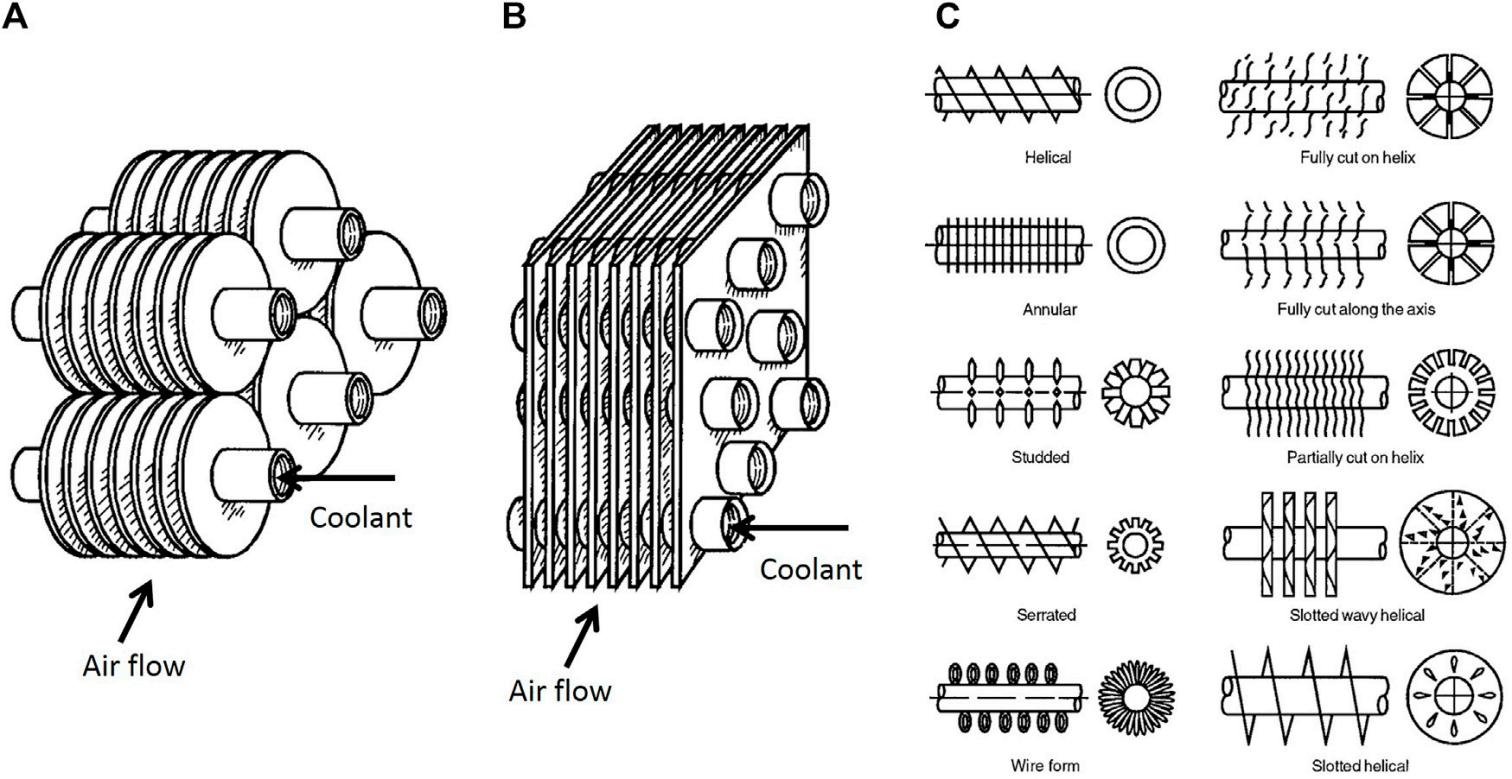
**(A)** Individually finned tubes; **(B)** Flat (continuous) fins on an array of tubes; **(C)** Individually finned tubes (Zhuo et al., 2018).

Ice formation (frost) on the surface of evaporators is a common and undesired phenomenon, which remarkably reduces the system efficiency. Figure 10 shows two types of the most used conventional tube-fin evaporators in the HVAC industry. In these devices, the coolant flows inside the tubes, while the air flows through the fins. The formed ice layer on the fins deteriorates the heat transfer rate not only by acting as an insulator layer on the evaporator surface (tube surfaces) but also by blocking the air channel and increasing the flow resistance.

**FIGURE 11:**
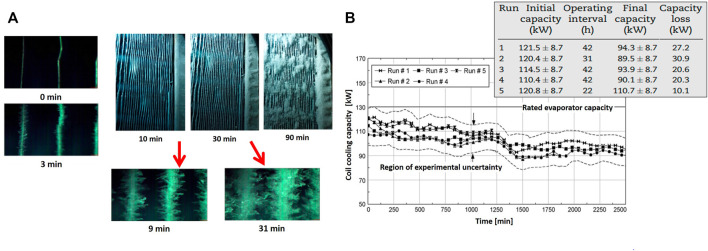
**(A)** Macroscopic and microscopic images of frost formation on the surface of evaporators; **(B)** Evaporator cooling capacity versus time as a function of ice layer thickness (Wang et al., 2019).

The frost formation on the evaporator is shown in Figure 11A. As seen, ice layers gradually cover the fin surfaces until they block the airflow passage. According to microscopic images, the ice crystals start to be visible between the fins at time *t* = 3 min. Here, some big ice crystals grow up to frost branches. At time *t* = 9 min, the crystals start to form larger units. However, these branches are not large enough to pass over the fins. As crystals grow larger, the branches start to grow, which is shown at time *t* = 31 min. At this stage, the ice crystals touch the neighboring branches and form thick and high-density ice layers, which block the fin surface. Figure 11B shows the evaporator cooling capacity as a function of time for the five different conditions. The evaporator capacity deteriorates with operating time. The accumulated ice increases the flow resistance and associated air side pressure drop. With a decrease in the airflow rate, the temperature drop decreases along with the evaporator, which leads to deterioration in the cooling capacity of the evaporator with time.

### Aviation

**FIGURE 12:**
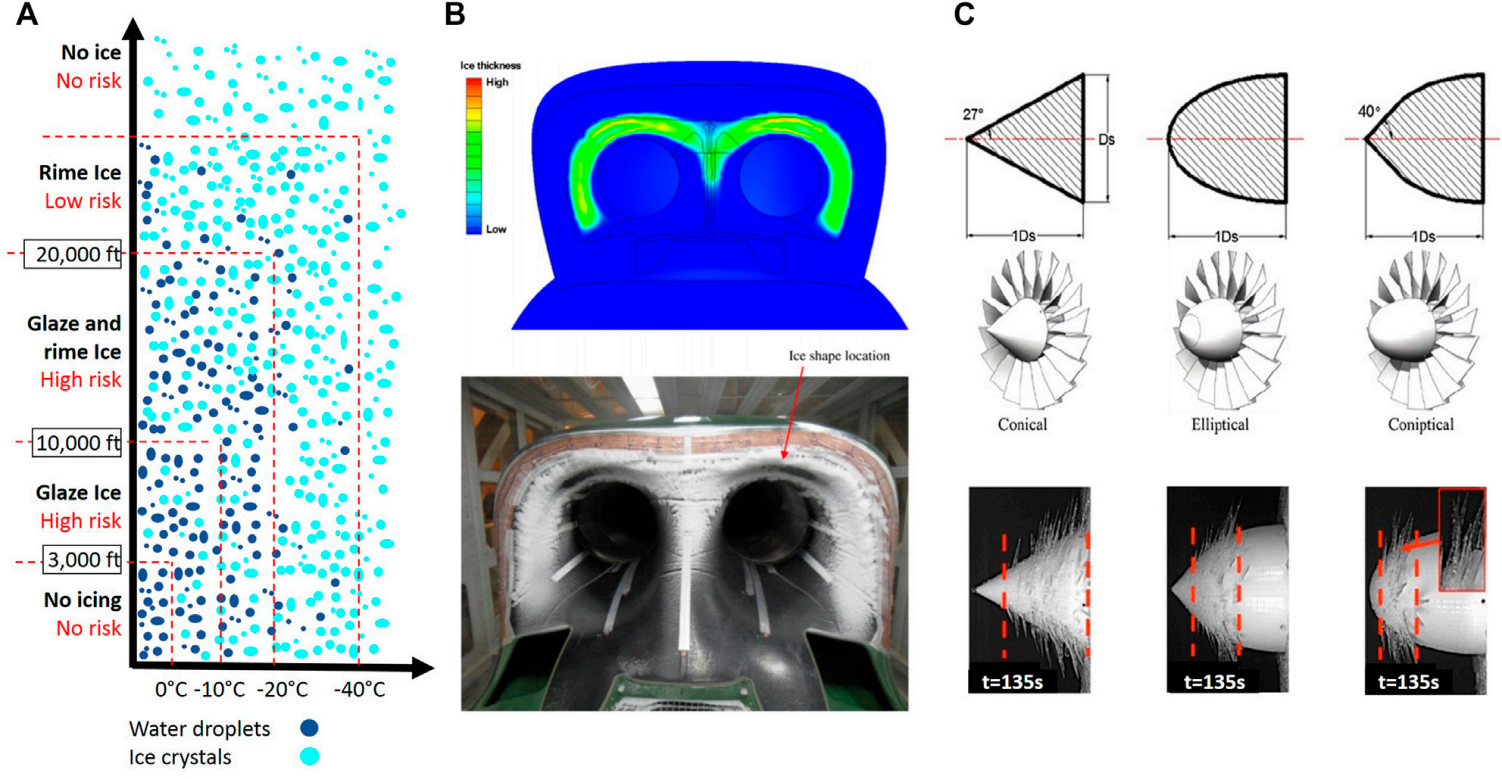
**(A)** Risk of icing vs. altitude; **(B)** numerical and experimental results on icing in air flow intake; **(C)** effect of propeller shape on icing and resultant ice formation (Dalili et al., 2009; Gurumukhi et al., 2020).

The degree of icing depends on the height above the ground level. Icing types include rime ice, glaze ice, and mixed ice, which is a mixture of rime ice and glaze ice. Rime ice is nonhomogenous, consisting of powdery ice particles, shaped into finger-like structures. It is soft and less dense and happens at very low temperatures when 100% impinging droplets freeze. On the other hand, Glaze ice is a homogenous transparent hard continuum solid, and it is harder and denser. Glaze ice happens close to the freezing temperature when the freezing fraction of impinging droplet is not 100% and some droplets run along the blade surface as a very thin water film. The concentration of water droplets and ice embryos changes with altitude. The risk of icing is high at altitudes up to 10,000 feet. Ice formation on the surface of aircraft including wings, airfoils, and rotors might cause catastrophic problems (Figure 12A). Ice on rotors and blades hinders their performance and dynamic stability. Turbine engines are particularly vulnerable against icing because ice formation in the air intake area restricts the air inflow, and the separated pieces of ice might cause structural damages by being drawn into the engine (Figure 12B). During the flight, supercooled water droplets impact the aircraft surface and rapidly form ice layers. The accumulated ice layers change hydrodynamics around the wings or rotors and increase the drag. An airfoil drag might increase up to 200 percent, which might lead to the loss of lift force (Wei et al., 2020) (Zhao et al., 20092009) (Shah and Sekulic, 2003). Power reduction and engine roughness are the first signs of ice formation on propellers or spinners. The unevenly accumulated ice causes instability in blades. The resulting vibration puts excessive pressure on blades and motor bearings, leading to a possible failure. Icing-induced airflow disturbance near the wings and tail is more important than its weight. By changing the airfoil cross-section geometry, ice layers reduce the lift force while increasing the drag and stalling speed (Figure 12C). In this situation, full power and higher angle of attack are required to keep the altitude and to compensate for the degraded thrust. With a higher angle of attack, lower parts of the wing will start to form ice and add drag and weight to the aircraft.

During take-off and landing at sufficient speeds, a thin layer of ice might form on the windshield, which might cause frosting, thereby remarkably reducing the visibility of the runway during a critical time in flight. The structural surface with thin leading edges such as antennas, horizontal stabilizers, rudders, and landing gear struts are the first locations, which experience ice accumulation and result in faults in radio antennas, pilot tubes, and static vents. The thin air temperature gauge, altimeter, airspeed, climb rate, gyroscopic instruments, pilot tube, and static pressure ports are flight instruments, which might not operate correctly or cease operation due to ice accumulation. Not only all communications between VHF Omni-Directional Range reception and ground might be affected by ice, but the ship antennas might also break under the accumulated ice weight.

### Pavements

The freezing of water on the pavement surfaces causes slippery conditions, which will impair the safety of travel and transportation. This condition is of great importance in several applications such as airport runways, railways roads, and highways. Although de-icing chemicals and agents are mostly being used as a solution to reduce the risk of casualties, a significant problem is wastewater originating from de-icing pavements. Airport operators worldwide are under scrutiny to cope with the regulations focusing on the environmental impacts of de-icing practices. Chemical substances used for winter maintenance of airports are the main source of water and land pollution. After use, most of the de-icing and anti-icing agents get typically mixed with stormwater runoff and enter ground and water near the airfield pavements (Bowden, 1956).

**FIGURE 13:**
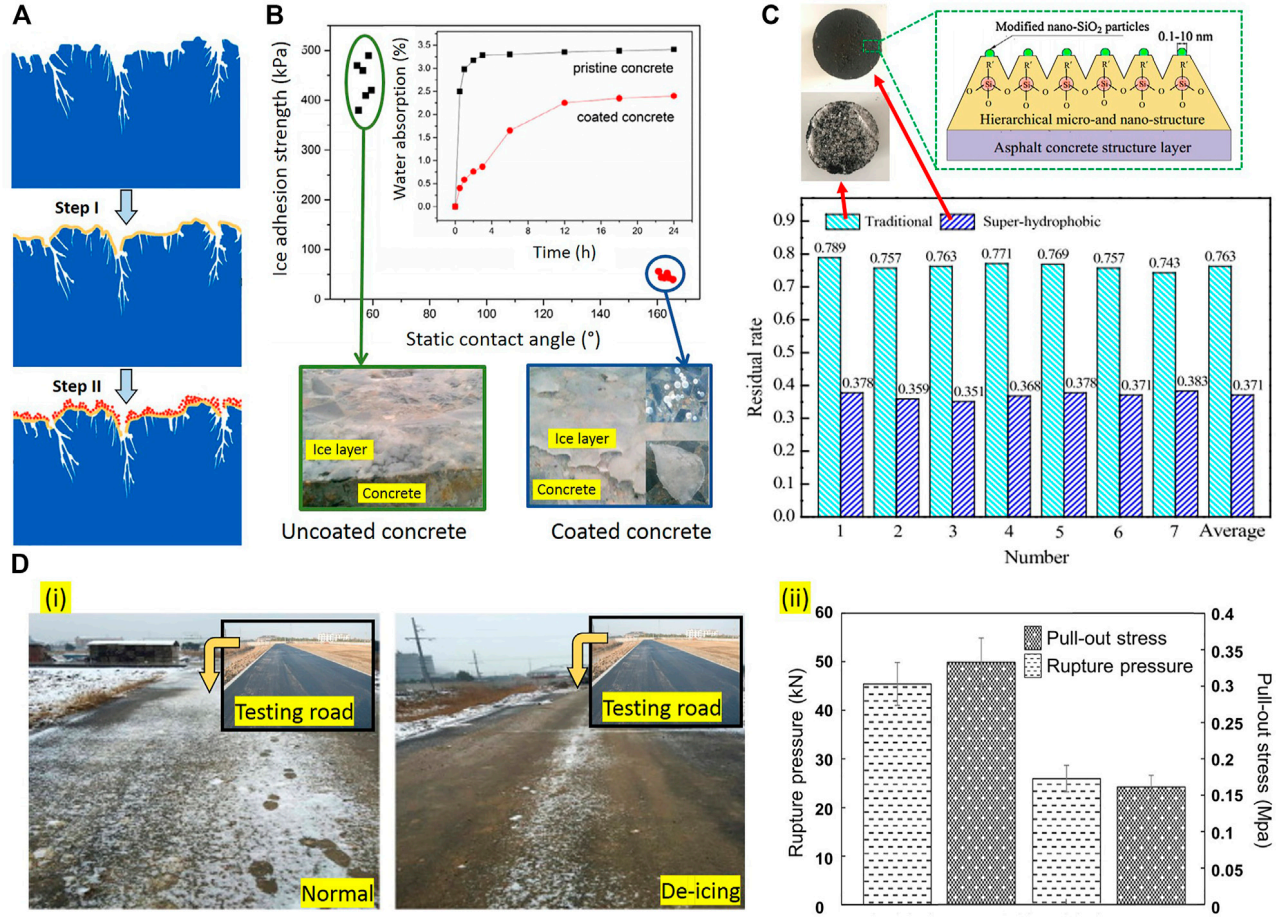
**(A)** Two-step anti-icing/superhydrophobic/ice-phobic coating method; **(B)** The water contact angles and Ice adhesion strength of uncoated and superhydrophobic coated concrete (Gurumukhi et al., 2020); **(C)** Residual rates of ice in the freezing-rain environment for traditional and superhydrophobic coated concrete (Dalili et al., 2009); **(D)** Ice layer pull out and rupture tests on deicing asphalt **(i)** and observation during snowing weather on normal asphalt pavement and deicing asphalt pavements **(ii)** (Kimura et al., 2003).

Spraying superhydrophobic/icephobic materials is the widely used method to decrease surface energy. A two-step coating method (step I: spraying the adhesion layer, step II: spraying the superhydrophobic material) can be applied to increase the adhesion of such coatings (Figures 13A,B). The ice residual rate $\left(m_{frozen}/\left(m_{frozen}-m_{rolling-ice}\right)\right)$ can be used to show the effectiveness of the coating. The freezing rain residual rate for superhydrophobic specimens is much lower than the traditional concrete specimens, which is an indication of the lower ice adhesion on the treated surface. In other words, the superhydrophobic coated surfaces can remarkably inhibit the formation of ice on the road (Figure 13C). The utilization of anti-icing agents in the form of the mixture could also enhance the snow melting process of asphalt pavements. In this method, upon contact with snow, the deicer melts down the snow and formulates a deicing solution on the pavement surface. Afterwards, the solutions additionally facilitate the release of deicers, which further accelerates snow melting and prevents ice accumulation on the asphalt pavement (Figure 13D). The larger rupture pressure and lower pull-out stress of modified asphalt suggest that the ice layer on the normal asphalt is much stronger than that on the modified asphalt, and the bond between ice and deicing asphalt mixture is much weaker than that between ice and normal asphalt mixture (Lv et al., 2014).

## Durability, Economic Aspect, and Large-Scale Production of APFs

### Durability of AFPs

The stability and durability of the proteins are determined by their folded structure formed from the interactions between different amino acid residues within their primary structures. Generally, the overall stability of proteins depends on their core subunit interactions within tertiary and quaternary structures which are defined by various bindings such as hydrophobic interactions, van der Waals, electrostatic, and disulfide bridges (Gromiha and Gromiha, 2010). The dynamics of the protein conformation is subject to change by the intrinsically free energy between entropy and enthalpy for both folded and unfolded configurations. However, studies suggested that the stability of a folded protein is significant due to its overall residual entropy (Bigman and Levy, 2020). Parameters such as temperature and pH change the protein stability and cause denaturation as a result of destabilizing bonds and altering charges. Thermostable proteins that are highly stable at 100°C are reported to be predominant in hydrophobic residues, including valine leucine and isoleucine, and the higher number of disulfide bridges (Gharib et al., 2016; Gharib et al., 2020).

Antifreeze proteins showed thermodynamic stability in a wide range of temperatures. Like other proteins, depending on their amino acid compositions and folded structures, they are subject to refolding or retaining their activity once exposed to different temperatures (García-Arribas et al., 2007). The stability of these remarkable polypeptides in various temperatures might result from low thermodynamics but high kinetic stability (Friis et al., 2014). However, the heat-stable antifreeze protein from *perennial ryegrass*, Lolium perenne, remained intact at the boiling temperature and demonstrated higher ice recrystallization inhibition and lower antifreeze as compared to its counterpart in antarctic fish. This protein has a unique sequence of amino acids with minimum homology with other antifreeze proteins and exhibited extremely hydrophilic properties abundant in asparagine, serine, and valine and less number of residues with hydrophilic or aromatic side chains (Sidebottom et al., 2000). The protein stability at various temperatures depends on their relevant colloidal stability, defined by structural ability to refold, and therefore evades the aggregation (Chi et al., 2003). In antifreeze proteins, two thermodynamic factors determine the colloidal stability at various temperatures. First, the cysteine or disulfide bridges contribute to the rigidity of protein towards heat. Second, the widths of the ice-binding motifs play a vital role in their stability. The AFPs with the higher melting temperature are shown to have a higher number of disulfide contents and lower motifs. Therefore, antifreeze proteins with irreversible and unfolded properties at higher temperatures are likely to lack disulphide bridges. The antifreeze protein from *Rhagium mordax,*RmAFP1, with melting temperature (T_m_) at 28.5°C (pH 7.4) with one disulfide, was shown to have kinetic stability beyond its melting temperature and ability to revert to its native state at 70°C (García-Arribas et al., 2007; Friis et al., 2014). Similarly, the antifreeze protein from desert beetle, *D. Canadensis* with T_m_ 84°C remained with no activity changes once exposed to a much higher temperature at 100°C (Li et al., 1998). On the contrary, the *P. americanus* with dimeric structure lost its activity due to a complete denaturation of the tertiary and quaternary structure once exposed to room temperature (22°C) (Marshall et al., 2005).

Nevertheless, since AFPs evolved with different structural integrity and there is a lack of sequence homology among their various types, they are likely to exhibit different characteristics in their physical properties (Barrett, 2001). Similar to other proteins based on their surrounding buffer composition, the stability and thermotolerance properties of AFPs are highly prone to alter (Salvay et al., 2007).

### Large Scale Production of AFPs

During recent years, the global demand for AFPs has increased substantially due to the rise in the consumption of frozen food. Using these naturally produced proteins is considered a sustainable and safe way to store various products and to facilitate their trading while maintaining food quality throughout the process. Applying ice nucleation proteins to certain delicate foods and fruits is a promising scheme to preserve their properties at suitable temperatures for a more extended period. In the presence of these remarkable proteins, the temperature, at which ice nucleation usually occurs, increases, resulting in mitigation of the freezing time and minimizing the size of the ice crystals (Ye et al., 2020). Hence, higher temperature maintenance requires less energy. In the agricultural industry, using the transgenic AFP producing plants promotes geological growth for certain crops such as wheat or potato by extending their growing seasons (Cutler et al., 1989). In the ice-cream manufacturing process, the crystallization inhibitory effect of the AFPs is exploited to conserve the size of the crystals during the production and storage period (Lv et al., 2014). Similarly, in the meat industry, the injection of antifreeze glycoproteins to the meat products before the freezing process contributes vastly to prevent the early denaturation and therefore increases the product’s shelf-life and brings no additional taste unlike the other cryoprotective substances (Provesi et al., 2016). In the fish industry, the winter flounder antifreeze protein inhibits potential ice growing phenomena in the harvested fish during transportation that consequently promotes energy storage and economic development (Cutler et al., 1989). In the bakery industry, using recombinant yeast, *Saccharomyces cerevisiae*, with an anti-freezing activity enhanced the dough cold tolerance and gassing rate quality which eventually led to the production of high-quality dough texture (Xu et al., 2009).

Antifreeze proteins are important antifreeze materials that have been widely used in the industry. The economic impact of using antifreeze proteins is significant, and therefore over the last decades, they have been studied extensively to optimize their large-scale production from various sources of organisms for improvement in various applications (Eskandari et al., 2020). Genetic engineering combined with large-scale culturing has succeeded in the production of commercial quantities of several proteins such as cold temperature active enzymes used in laundry detergents, diagnostic antibodies, and pharmaceutical proteins (Nomoto, 1993). Although highly purified proteins are crucial for research purposes, even poorly purified samples are satisfactory for industrial and commercial usage. Today, enzymes being used in food processing are the partially purified products of the cultivation. Similarly, due to the functionality of AFPs in solutions containing a large number of impurities, partially purified antifreeze proteins can be still used in the food industry. This means that the highly purified AFPs are not necessary to be used in all technologies and industries utilizing AFPs (Nishimiya et al., 2008).

## Future Research Directions and Conclusion

The naturally occurring antifreeze proteins (AFPs) are viable for industrial-scale production, and these bio-based materials are sought in the industry from sustainability and environmentally friendly aspects. Although the development of the coating of the AFPs on industrial surfaces is adaptable i.e., dip coating (Charpentier et al., 2013), the temperature might quickly denature the proteins. Not only the stability of AFPs should be maintained, but also the thermal denaturation should be prevented when used in industrial applications. For instance, trehalose coating can be used to enhance the stability of proteins on metallic surfaces. Trehalose is an essential compound for maintaining the hydration of the surface, which is vital for protein protection (Gwak et al., 2015). There are several numbers of potentially protective shields that may alternatively be used to preserve the biocoated surfaces. The biopolymers such as chitin or chitosan, the main composition of the arthropod’s exoskeleton, are alternative shields in this regard.

This review discusses various sources of antifreeze proteins, their potential advantages, and their need for industrial applications. We reviewed different approaches to implement these highly active polypeptides to form a futuristic applicable coated material. What makes these macromolecules highly suitable is their biocompatibility, low cost, and scalability, in addition to their environmentally friendly nature, which is aligned with the current global demand. Although coating and immobilization of AFPs on engineering surfaces may be a challenge for adaptation of such technology for energy applications, promising laboratory results are reported in the relevant industry. The effective coating of AFPs using metal-associated polypeptides on various industrial equipment such as refrigerators and wind turbines guarantees future advancement in energy conservation and saving. Among the suggested methods of antifreeze coating, what is most noticeable is based on simple molecular biology techniques to alter and clone the gene encoding for these proteins using microbial sources attached to the metal-binding peptides. The infinite microbial source ensures a convenient, biocompatible, and controllable approach for large-scale production. Biobased materials such as trehalose and chitin that are recommended for the protection of the coated surface could be also generated *via* microbes.

## Abbreviations

AFGP, Antifreeze Glycoprotein; AFP, Antifreeze Protein; IBP, Ice binding protein; INP, Ice Nucleation Protein; TH, Thermal Hysteresis.
